# Genetic Risk Factors in Lupus Nephritis and IgA Nephropathy – No Support of an Overlap

**DOI:** 10.1371/journal.pone.0010559

**Published:** 2010-05-10

**Authors:** Mai Tuyet Vuong, Iva Gunnarsson, Sigrid Lundberg, Elisabet Svenungsson, Lars Wramner, Anders Fernström, Ann-Christine Syvänen, Lieu Thi Do, Stefan H. Jacobson, Leonid Padyukov

**Affiliations:** 1 Rheumatology Unit, Department of Medicine, Karolinska Institutet, Stockholm, Sweden; 2 Department of Nephrology, Danderyd Hospital, Karolinska Institutet, Stockholm, Sweden; 3 Department of Internal Medicine, Hanoi Medical University, Hanoi, Vietnam; 4 Nephrology Unit, Department of Medicine, Karolinska University Hospital, Stockholm, Sweden; 5 Transplantation Center, Sahlgrenska University Hospital, Göteborg, Sweden; 6 Department of Nephrology, Linköping University Hospital, Linköping, Sweden; 7 Department of Medical Sciences, Uppsala University, Uppsala, Sweden; Health Canada, Canada

## Abstract

**Background:**

IgA nephropathy (IgAN) and nephritis in Systemic Lupus Erythematosus (SLE) are two common forms of glomerulonephritis in which genetic findings are of importance for disease development. We have recently reported an association of IgAN with variants of *TGFB1*. In several autoimmune diseases, particularly in SLE, *IRF5*, *STAT4* genes and *TRAF1-C5* locus have been shown to be important candidate genes. The aim of this study was to compare genetic variants from the *TGFB1*, *IRF5*, *STAT4* genes and *TRAF1-C5* locus with susceptibility to IgAN and lupus nephritis in two Swedish cohorts.

**Patients and Methods:**

We genotyped 13 single nucleotide polymorphisms (SNPs) in four genetic loci in 1252 DNA samples from patients with biopsy proven IgAN or with SLE (with and without nephritis) and healthy age- and sex-matched controls from the same population in Sweden.

**Results:**

Genotype and allelic frequencies for SNPs from selected genes did not differ significantly between lupus nephritis patients and SLE patients without nephritis. In addition, haplotype analysis for seven selected SNPs did not reveal a difference for the SLE patient groups with and without nephritis. Moreover, none of these SPNs showed a significant difference between IgAN patients and healthy controls. *IRF5* and *STAT4* variants remained significantly different between SLE cases and healthy controls. In addition, the data did not show an association of *TRAF1-C5* polymorphism with susceptibility to SLE in this Swedish population.

**Conclusion:**

Our data do not support an overlap in genetic susceptibility between patients with IgAN or SLE and reveal no specific importance of SLE associated SNPs for the presence of lupus nephritis.

## Introduction

Several common gene variations have recently been shown to associate with different autoimmune diseases, particularly Systemic Lupus Erythematosus (SLE). Some nucleotide polymorphisms (SNPs) have been shown to associate with single autoimmune disease, while other SNPs associate with several diseases. Interferon regulatory factor 5 *(IRF5)* polymorphism has been shown to be a risk factor for the development of SLE [1], [2], [3], [4], [5], rheumatoid arthritis (RA) [6], [7], [8], [9], multiple sclerosis (MS) [10], Sjögren's syndrome [11] and inflammatory bowel disease [12]. Signal transducers and activator of transcription 4 *(STAT4)* and TNF receptor-associated factor 1-Complement component 5 (*TRAF1-C5*) polymorphisms have been found to associate with both SLE and RA [13], [14], [15], [16]. Recently we reported that transforming growth factor-β1 *(TGFB1)*, an important cytokine gene, is in association with IgA nephropathy (IgAN) [17].

Immunological and biochemical similarities between SLE and IgAN demonstrate a direct link to impaired immune function in both diseases [18]. Patients with lupus nephritis and IgAN both have circulating immune complexes and display anti-C1q antibodies, which might point to certain pathogenic similarities in these glomerular disorders [18], [19]. Moreover, lupus nephritis and IgAN are both chronic renal diseases that are classified in the “predominant” inflammatory group, based on morphological similarities [20], [21]. We hypothesized that it may be an overlap in genetic susceptibility between lupus nephritis and IgAN and that there could be specific genetic makers associated to the development of nephritis in SLE patients.

To test this hypothesis we compared the genotype, allelic and haplotype frequencies from *IRF5*, *STAT4* and *TRAF1-C5* polymorphisms between IgAN patients and healthy controls, and SLE patients with and without nephritis, from *TGFB1* polymorphisms between SLE patients and healthy controls.

## Materials and Methods

### Patients and healthy subjects

Two cohorts of patients with SLE or IgAN, altogether 1252 individuals, were included in the present study. The cohort of patients with SLE, consisted of 272 SLE patients, all self-reported Caucasians from 18 to 80 years of age (mean age 45±14 years). 106 SLE patients had biopsy proven nephritis (39%) and 166 SLE patients had no clinical or laboratory signs of nephritis (61%). The control group for SLE patients consisted of 307 healthy age- matched individuals from the same population in Sweden, who were 17 to 70 years old, mean age 44±13 years.

In the IgAN cohort, there were altogether 673 DNA samples, of which 196 samples were obtained from patients with biopsy- proven IgAN, all self-reported Caucasians, and 477 samples were collected from gender- and age matched healthy controls from the same population in Sweden. Patients with Henoch-Schönlein purpura or with other forms of glomerulonephritis and individuals with self-reported non-Caucasian ancestry were excluded in our study. All patients gave written informed consent and the study was approved by the Ethics Committee of the Karolinska University Hospital, Stockholm, Sweden.

### DNA extraction, selection of genetic markers, and genotyping

DNA was extracted from EDTA blood samples (5–10 ml) by the “salting out” method, as described elsewhere [22]. The SNPs were selected because they had previously been shown to be associated with SLE [5], [14], [15], RA [9], [13], [15], or with IgAN [17]. The SNPs were genotyped by fluorescent single base extension using the multiplex SNPstream system (Beckman Coulter Inc) or by TaqMan allelic discrimination assay (Applied Biosystems, Foster City, U.S.A) (Table 1). All analyzed SNPs were in Hardy-Weinberg equilibrium and the average positive rate of the genotype detection was 97.2%. DNA samples with poor performances in genotyping (<95% successful genotypes) were excluded from the statistical evaluation.

**Table 1:** Polymorphisms of *TGFB1*, *IRF5*, *STAT4* genes, *TRAF1-C5* locus in the study.

Gene	SNP	Position	Chromosome	Chromosome position	Alleles	Methods
*STAT4*	rs10181656	Intron 3	2q32.2-q32.3	183829287	C/G	TaqMan
*IRF5*	rs729302	Promoter	7q32	128356196	A/C	SNPstream
*IRF5*	rs4728142	Promoter	7q32	128361203	A/G	SNPstream
*IRF5*	rs2004640	The intron-exon border of exon 1B	7q32	128365537	G/T	SNPstream
*IRF5*	rs3807306	Intron 1	7q32	128367916	G/T	SNPstream
*IRF5*	rs10954213	3′ UTR	7q32	128376663	A/G	SNPstream
*IRF5*	rs11770589	Exon 10 3′ UTR	7q32	128376724	A/G	SNPstream
*IRF5*	rs2280714	3′flanking region	7q32	128381961	C/T	SNPstream
*TRAF1-C5*	rs3761847	5′ flanking region	9q33-q34	93307696	A/G	TaqMan
*TGFB1*	rs6957	Downstream 3′genomic region	19q13.1	46522446	C/T	TaqMan
*TGFB1*	rs2241715	Intron 1	19q13.1	46548726	G/T	TaqMan
*TGFB1*	rs1982073	Signal sequence of exone 1	19q13.1	46550761	C/T	TaqMan
*TGFB1*	rs1800469	Promoter	19q13.1	46552136	A/G	TaqMan

### Statistical analysis

To assess genotype, allele and haplotype frequencies, Pearson Chi-square and/or Fisher's Exact Tests were performed when appropriate with PASWStatistics 18.0 Software. Haplotype analysis was carried out by HaploView [23]. Power calculation was performed for two-tail or one-tail tests when appropriate for 5% threshold of significance.

## Results

### *IRF5*, *STAT4* and *TRAF1-C5* polymorphisms did not show an association with susceptibility and/or severity of IgAN

We did not find an association with disease susceptibility for the investigated SNPs in *IRF5*, *STAT4* genes and *TRAF1-C5* locus, in both the co-dominant model and the reccessive/dominant model, comparing patients with IgAN and healthy controls. One *IRF5* SNP (rs12539741) showed a difference between genotype distribution in IgAN patients and healthy controls in males in the dominant C model (p = 0.04). However, this association was not significant after Bonferroni correction for multiple comparisons. Comparing the allele and haplotype frequencies, we did not observe any significant differences for the SNPs between IgAN patients and controls (Table 2).

**Table 2:** Allelic frequencies of *IRF5*, *STAT4* and *TRAF1-C5* polymorphisms in IgAN patients and controls.

Gene	SPNs	Association Allele	Control/PatientRatio Counts	Control/Patient Frequencies	Chi Square	P Value*
*STAT4*	rs10181656	A	532∶388, 206∶178	0.578, 0.769	1.927	0.2
*IRF5*	rs729302	A	629∶299, 257∶123	0.678, 0.676	0.003	0.9
*IRF5*	rs4728142	G	505∶425, 206∶176	0.543, 0.539	0.015	0.9
*IRF5*	rs2004640	G	444∶486, 169∶207	0.477, 0.449	0.840	0.4
*IRF5*	rs3807306	G	455∶471, 177∶201	0.491, 0.468	0.574	0.4
*IRF5*	rs10954213	G	342∶584, 135∶241	0.369, 0.359	0.122	0.7
*IRF5*	rs11770589	G	475∶453, 193∶191	0.512, 0.503	0.093	0.8
*IRF5*	rs2280714	C	294∶636, 112∶266	0.316, 0.296	0.494	0.5
*TRAF1-C5*	rs3761847	C	739∶197, 297∶89	0.790, 0.769	0.651	0.4

*Uncorrected.

### *TGFB1* polymorphisms did not show an association with susceptibility to SLE or to lupus nephritis

No significant differences in genotype distribution or allele frequencies were observed between SLE patients and healthy controls for four investigated SNPs from the TGFB1 gene. In addition, there was no significant difference between lupus nephritis and healthy controls in both the co-dominant model and the recessive/dominant model of genotype frequencies and allelic frequencies (Table 3). *TGFB1* polymorphisms in selected SNPs did not show an association with SLE or with lupus nephritis among SLE patients.

**Table 3:** Allelic frequencies of *STAT4*, *IFR5*, *TRAF1-C5* and *TGFB1* polymorphisms in SLE patients and controls.

Gene	SPNs	Association Allele	Control/PatientRatio Counts	Control/PatientFrequencies	Chi Square	P Value*
*STAT4*	rs10181656	C	481∶127, 343∶201	0.791, 0.631	36.363	1.64E-09
*IRF5*	rs729302	C	124∶248, 105∶285	0.333, 0.269	3.722	0.0537
*IRF5*	rs4728142	G	206∶166, 160∶230	0.554, 0.410	15.708	7.39E-05
*IRF5*	rs2004640	G	181∶191, 134∶256	0.487, 0.344	16.048	6.17E-05
*IRF5*	rs3807306	G	177∶195, 139∶251	0.476, 0.356	11.182	8.00E-04
*IRF5*	rs10954213	G	131∶241, 102∶288	0.352, 0.262	7.364	0.0067
*IRF5*	rs11770589	A	195∶177, 203∶187	0.524, 0.521	0.01	0.9
*IRF5*	rs2280714	C	115∶257, 87∶303	0.309, 0.223	7.239	0.007
*TRAF1-C5*	rs3761847	A	334∶258, 277∶253	0.564, 0.523	1.946	0.2
*TGFB1*	rs6957	T	511∶89, 453∶91	0.852, 0.833	0.772	0.4
*TGFB1*	rs2241715	G	187∶419, 164∶378	0.309, 0.303	0.048	0.8
*TGFB1*	rs1982073	T	224∶384, 198∶344	0.368, 0.365	0.012	0.9
*TGFB1*	rs1800469	A	191∶417, 162∶378	0.314, 0.300	0.269	0.6

*Uncorrected.

### Genetic variations associated with susceptibility to SLE did not correspond to a specific association with lupus nephritis

To determine if SLE-related genetic variants associate specifically with nephritis in SLE patients, we genotyped up to nine variants from the *IRF5*, *STAT4* genes and the *TRAF1-C5* locus. We compared genotype frequencies in both the co-dominant model and recessive/dominant model between patients with lupus nephritis and SLE patients without nephritis. We detected no significant differences between these two groups. Moreover, there were no significant differences in allele frequencies of any investigated SNPs (Table 4). We found no differences in haplotype analyses in patients with SLE and healthy controls or between patients with lupus nephritis and SLE patients without nephritis.

**Table 4:** Allelic frequencies of *STAT4*, *IFR5*, *TRAF1-C5* and *TGFB1* polymorphisms in lupus nephritis against SLE patients without nephritis.

Gene	SPNs	Association Allele	Lupus without nephritis/Lupus nephritisRatio Counts	Lupus nephritis/none-nephritisFrequencies	Chi Square	P Value*
*STAT4*	rs10181656	C	210∶122, 133∶79	0.633, 0.627	0.015	0.9
*IRF5*	rs729302	C	65∶165, 40∶120	0.283, 0.250	0.510	0.5
*IRF5*	rs4728142	G	97∶133, 63∶97	0.422, 0.394	0.306	0.6
*IRF5*	rs2004640	G	87∶143, 47∶113	0.378, 0.294	2.988	0.1
*IRF5*	rs3807306	G	87∶143, 52∶108	0.378, 0.325	1.167	0.3
*IRF5*	rs10954213	G	65∶165, 37∶123	0.283, 0.231	1.289	0.3
*IRF5*	rs11770589	G	113∶117, 74∶86	0.491, 0.462	0.314	0.6
*IRF5*	rs2280714	C	59∶171, 28∶132	0.257, 0.175	3.618	0.06
*TRAF1-C5*	rs3761847	A	154∶166, 99∶111	0.481, 0.471	0.049	0.8

*Uncorrected.

## Discussion

This is the first investigation to study the importance of *IRF5*, *STAT4* and *TRAF1-C5* gene polymorphisms in patients with IgAN. Our data show no evidence of an association of these genes with the development of IgAN or distinct risk alleles for lupus nephritis in this Swedish population.

According to recent findings, the *IRF5* gene is an important candidate gene in different chronic diseases, especially systemic diseases related to inflammation and autoimmunity. A meta-analysis which included 15 studies regarding *IRF5* gene polymorphism and SLE, confirmed the importance of rs2004640 for SLE susceptibility [4]. *TRAF1-C5* and *STAT4* polymorphisms have been shown to associate with RA and SLE, and also with some other autoimmune diseases [5], [14], [15].

Thus, one might speculate that susceptibility to IgA nephropathy may be due to common variations in *IRF5*, *TRAF1-C5* and *STAT4* genes. Our data do however not confirm this hypothesis. Since no single marker (for *IRF5*, *TRAF1-C5* and *STAT4*), no haplotype associations (for *IRF5*) were detected in patients with IgAN, it is reasonable to rule out a strong influence of these gene polymorphisms in IgAN development or disease progression. We noticed that we have almost 80% power to detect a 10% difference in minor allele frequency (MAF) in our cases and controls. However, due to limited sample size, we cannot exclude minor influences from the investigated gene polymorphism on IgAN, which may also differ in different populations. On the other hand, *TGFB1* polymorphisms were found previously in association with the susceptibility to IgAN [17] but did not show any association with SLE or lupus nephritis in the present study.

There are immunological and biochemical similarities between lupus nephritis and IgAN, and both conditions are associated with immune complex formation and mesangial immune deposits. There are also a number of reports on patients with SLE who develop IgAN [24], [25]. However, there was no overlap in genetic risk factors in the here studied genes between SLE and IgAN patients or any specific genetic variants detected comparing lupus patients with or without nephritis.

An association of *TRAF1-C5* locus with SLE was recently detected in a relatively small cohort [14]. However, our data did not show an association between *TRAF1-C5* polymorphism neither in SLE nor in IgAN.

In conclusion, the findings in the present study do not support an overlap in genetic susceptibility between Swedish patients with IgAN or SLE and reveal no specific importance of SLE associated SNPs for presence of lupus nephritis.
